# When do co-infections matter?

**DOI:** 10.1097/QCO.0000000000000447

**Published:** 2018-04-26

**Authors:** Andrew J. McArdle, Anna Turkova, Aubrey J. Cunnington

**Affiliations:** a Paediatric Infectious Diseases, St George's Hospital; b MRC Clinical Trials Unit, Institute of Clinical Trials and Methodology, University College London; c Section of Paediatrics, Imperial College, London, UK

**Keywords:** co-infection, diagnosis, interactions, pathogenesis, susceptibility, treatment

## Abstract

**Purpose of review:**

Advances in diagnostic methods mean that co-infections are increasingly being detected in clinical practice, yet their significance is not always obvious. In parallel, basic science studies are increasingly investigating interactions between pathogens to try to explain real-life observations and elucidate biological mechanisms.

**Recent findings:**

Co-infections may be insignificant, detrimental, or even beneficial, and these outcomes can occur through multiple levels of interactions which include modulation of the host response, altering the performance of diagnostic tests, and drug–drug interactions during treatment. The harmful effects of chronic co-infections such as tuberculosis or Hepatitis B and C in association with HIV are well established, and recent studies have focussed on strategies to mitigate these effects. However, consequences of many acute co-infections are much less certain, and recent conflicting findings simply highlight many of the challenges of studying naturally acquired infections in humans.

**Summary:**

Tackling these challenges, using animal models, or careful prospective studies in humans may prove to be worthwhile. There are already tantalizing examples where identification and treatment of relevant co-infections seems to hold promise for improved health outcomes.

## INTRODUCTION

Globally, co-infections are almost certainly the norm rather than a rare curiosity. We are continuously exposed to multiple potential pathogens, most people are chronically or latently infected (be it with herpes viruses, helminths, or tuberculosis), and we all carry potential pathogens in our colonizing microbial flora. This means that nearly every new incident infection is likely to constitute some sort of co-infection. Nevertheless we know relatively little about which combinations of co-infections matter the most for our health. Here, using examples from the recent literature, we illustrate situations in which co-infections have important implications, both harmful and beneficial, and explain why it is sometimes difficult to be sure. 

**Box 1:**
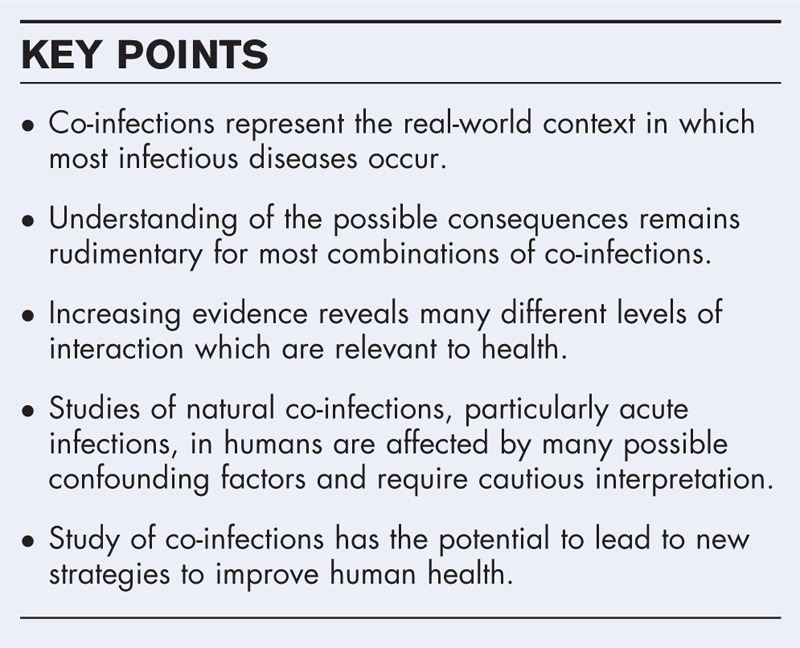
no caption available

## DOUBLE TROUBLE?

**FIGURE 1:**
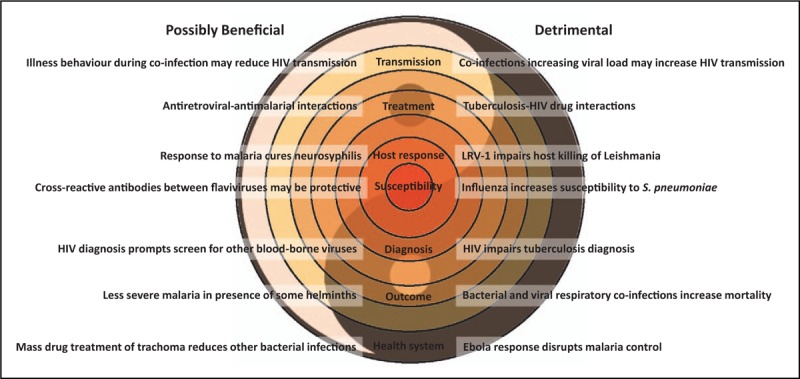
The good and the bad of co-infections. Co-infections can effect health through interactions at multiple levels. Examples are given where these interactions may be detrimental or sometimes beneficial. LRV-1, Leishmania RNA virus-1.

One might expect that infection with two or more pathogens would always be worse than infection with one. Even if co-infection is just bad luck, the adverse effect on health might be expected to be additive. But interactions do occur, on many levels, and these are not always detrimental (Fig. 1). Three examples involving malaria illustrate this well. Deliberate malaria co-infection (so-called, malaria therapy) was used as a treatment for neurosyphilis in the preantibiotic era, and is thought to have been moderately effective because of antitreponemal effects of the fever and cytokine response to malaria [1]. There is evidence that some helminth infections reduce malaria severity, possibly through immunomodulation [2]. Somewhat unexpectedly, antiretroviral therapy (ART) protected children with HIV from malaria, by prolonging the half-life of the antimalarial lumefantrine and effectively turning short courses of treatment into medium-term prophylaxis [3].

Unfortunately, determining the consequences of co-infection through observation of natural infections in humans is rife with problems because there are so many possible confounders. These include the presence of additional infections other than those being studied; the order, timing, and natural history of each infection; shared risk factors for acquisition of infections; and shared risk factors for their adverse outcomes. Interactions between infections which influence their likelihood of detection at earlier or later disease stages may also introduce bias. These challenges in human studies mean that co-infections are increasingly being investigated in animal models where conditions can be tightly controlled, and with careful consideration these can be useful to help to explain observations in humans.

## CO-INFECTIONS IN HIV

In people living with HIV (PLHIV) co-infections usually do matter, and most have adverse consequences. Despite providing some of the most obvious examples for every level of interaction illustrated in Fig. 1, HIV co-infection is really a special case because of lifelong infection and acquired immunodeficiency. The burden of co-infections in PLHIV is hard to quantify. Some insights come from intervention studies such as the recent randomised controlled trial of combined antifungal, antituberculous, antihelminthic, and antibacterial prophylaxis started at the time of ART initiation in African children and adults with profound immunosuppression. This regime prevented one death for every 30 patients treated in comparison to standard prophylaxis with cotrimoxazole alone over a 24-week period [4^▪▪^].

### HIV and tuberculosis

Tuberculosis is the leading cause of opportunistic infection and death among PLHIV. HIV is a potent risk factor for tuberculosis and complicates every aspect of tuberculosis care from prevention to diagnosis and treatment, whereas tuberculosis increases progression of HIV and contributes to slower CD4 recovery and faster virological failure on ART [5]. Recent work has shown tuberculosis incidence after ART initiation is significantly lower in PLHIV with CD4 more than 500 cells/μl compared to their counterparts with lower CD4 counts [6^▪^]. Ongoing HIV replication is an important risk factor for tuberculosis, regardless of CD4 cell counts [7^▪^], but tuberculosis risk does not differ before and after ART initiation when appropriately controlled for laboratory values and ART exposure [8^▪^].

Timely tuberculosis diagnosis is challenging in PLHIV because of high rates of smear negative and extrapulmonary tuberculosis. Clinical screening performs poorly in PLHIV and may miss up to 25% of all laboratory-confirmed tuberculosis cases and up to 70% among HIV-infected pregnant women [9^▪▪^]. Molecular and lateral flow diagnostics with greater sensitivity are showing promise for improving the situation [10,11^▪▪^,^12^,^13^^▪▪^,^14^].

Treatment of tuberculosis in PLHIV is challenging because of drug–drug interactions and overlapping toxicities with ART. Despite this, early ART initiation within the first 8 weeks of antituberculous therapy was associated with favourable outcomes in a large multinational cohort study in children [15^▪▪^]. Preventing tuberculosis in PLHIV is also complicated – although isoniazid preventive therapy (IPT) has been shown to be effective there are concerns that widespread use will drive the spread of isoniazid resistance. New estimates suggest that in the context of a declining/controlled tuberculosis epidemic, tuberculosis incidence and mortality benefits of continuous IPT for PLHIV outweigh the potential resistance risks [16^▪^]. A systematic review of universal IPT in children with no known tuberculosis exposure showed reduction of tuberculosis among children not receiving ART but, perhaps surprisingly, no clear benefit for children on ART [17^▪^].

### HIV and hepatitis B and C viruses

Viral hepatitis is associated with increased morbidity in PLHIV. End-stage liver disease is most common in patients with hepatitis B virus hepatitis C virus (HCV) HIV co-infection, then in dual infections, and much less common in HIV monoinfection [18^▪▪^]. Viral hepatitis is also associated with extrahepatic complications in PLHIV such as increased risk of non-Hodgkin lymphoma [19^▪^], kidney disease [20,21^▪^], osteoporosis and fractures [21^▪^], and more severe cognitive impairment [22]. Hepatitis virus co-infection also slows immunological recovery in pregnant women [23^▪^] and children [24^▪^] with HIV, and HCV contributes to an ongoing immune activation and immune dysfunction even in controlled HIV infection [25^▪^,^26^,^27^].

Directly acting antivirals now allow more than 95% HCV cure rates, regardless of HIV co-infection [28,29,30,31^▪^,32^▪^,^33^], and HCV eradication reduces mortality, HIV progression, liver-related events, and diabetes mellitus [21^▪^,^34^^▪▪^]. Well tolerated and effective regimes for PLHIV on ART are now achievable [35,36,37^▪▪^,^38^].

## BEYOND HIV

Chronic co-infections with HIV clearly demonstrate many potentially harmful impacts, but the evidence can be much less clear when acute infections are considered.

### Ebola–malaria co-infection

The 2014–2015 West African Ebola virus disease (EVD) epidemic ravaged countries which already suffered a high burden of malaria and bacterial infections. Differentiating EVD from other causes of febrile illness and identifying co-infections was problematic so pragmatic guidelines advised empirical antimalarial and antibiotic treatment [39]. Subsequent studies have tried to characterize the burden and consequences of co-infection. Of four large studies (albeit employing quite different methodologies), three concluded that malaria co-infection resulted in increased mortality in individuals with EVD [40^▪^,^41^^▪▪^,^42^], whereas one study concluded the opposite [43^▪^]. These discordant findings highlight some key challenges for studying acute co-infections.

Malaria-associated co-infections are particularly difficult to study because *Plasmodium* can cause repeated acute, chronic, and asymptomatic infections, and individuals in endemic countries develop a degree of naturally acquired immunity which accumulates over many years. Asymptomatic infection with *Plasmodium falciparum* is common in highly endemic settings, but in a febrile individual coinfected with an additional potential pathogen it is almost impossible to know whether *P. falciparum* detected in blood is the sole cause of illness, contributing to illness, or just a bystander. Higher parasite load and younger age generally associate with greater likelihood of symptomatic disease, allowing the attributable fraction of febrile illness because of malaria to be calculated at a population level by comparison with parasite loads detected in appropriately matched healthy community controls [44]. In contrast to *Plasmodium* infection, it is assumed that almost all individuals with Ebola virus infection will manifest EVD, and it remains controversial whether Ebola virus infection may produce minimal or no symptoms [39]. It is conceivable that presymptomatic EVD may be detected in an individual with malaria, particularly when there is active surveillance for febrile illness in EVD contacts. None of the four studies of EVD and *P. falciparum* co-infection had appropriate control groups to determine malaria attributable fractions of febrile illness, so they are all likely to be confounded by relationships between parasite load, age, and coincidence of exposure and comorbidities. However, the apparent protective effect of *P. falciparum* in one study led to the suggestion that malaria therapy might be used to treat EVD [45]. Although the other studies would caution against this, the urgent need for effective treatments against EVD makes it important to resolve the controversy and explore possible underlying biological mechanisms.

### Helminths and tuberculosis

Helminths are among the most prevalent pathogens globally. As they stimulate a type 2 helper T cell (Th2)-biased immune response, whereas protection from tuberculosis requires a type 1 helper T cell (Th1) response, the question has arisen whether co-infection may compromise defence against tuberculosis. In latent tuberculosis, co-infection with *Strongyloides stercoralis* reduced systemic and tuberculosis antigen-stimulated type 1 and type 17 cytokines, and increased systemic type 2 and regulatory cytokines [46^▪^]. Following treatment for *Strongyloides stercoralis*, type 1 and type 17 cytokine responses increased, along with increases in *Mycobacterium tuberculosis*-specific immunolgobulin M and immunoglobulin G [46^▪^,47^▪^]. However, real-world evidence that helminth-tuberculosis interactions are clinically important is less convincing. A large cross-sectional study of tuberculosis patients and uninfected household contacts in Tanzania, showed that tuberculosis infection was associated with *Schistosoma mansoni* infection, though this was just one of many helminths studied and the significance was borderline [48^▪^]. Interesting, and of greater statistical significance, was the finding that tuberculosis patients who did have *S. mansoni* infection had lower sputum bacterial loads, hinting at more complex interactions than those predicted from the Th1/Th2 paradigm. Consistent with this, *Mycobacterium bovis* bacterial loads were also decreased in cattle by co-infection with the fluke *Fasciola hepatica,* and co-infection was associated with reduced phagocytosis of mycobacteria [49^▪^].

Another practical concern is whether the presence of helminths may influence immune-based diagnostic tests for tuberculosis infection. Although there is some evidence that helminth infection reduces reactivity to purified protein derivative and increases the proportion of indeterminate interferon-γ release assay results in human tuberculosis, findings are far from conclusive [50]. However, in experimental bovine tuberculosis, co-infection with *Fasciola hepatica* reduced interferon-γ responses [49^▪^], consistent with earlier discovery of reduced intradermal purified protein derivative positivity, and estimates of a one-third reduction in ascertainment [51].

### Helminths and other co-infections

A recent public health success story in dealing with the challenge of co-infections comes from two neglected tropical infections: *Loa loa* and *Onchocercha volvulus*. Onchocerciasis is a common cause of blindness (so-called, river blindness), and the burden of disease can be reduced by mass administration of ivermectin. Yet mass treatment can cause severe encephalopathy in communities where there is also a high-burden of *Loa loa* filarial infections [52]. As a result, some of the worst affected communities have been excluded from mass-treatment programmes because of excessive risks. Automated video microscopy screening of blood samples to detect and quantitatively measure *Loa loa* burden allowed just over 2% of individuals to be excluded, and ivermectin treatment to be reintroduced without serious adverse events [53^▪▪^].

Helminth infection has also revealed an interesting perspective on the complexity of interactions occurring during co-infections. Rather than mediating its effects directly through modulation of the host immune response, *Heligmosomoides polygyrus* was found to modify colonization and virulence of *Salmonella* Typhimurium by modulating the mouse gut metabolome [54^▪^]. This suggests an intermediary role for the microbiota in the interaction between pathogens, and implies that current approaches to studying co-infections may be far too simplistic.

### Gastrointestinal and respiratory co-infections

Molecular diagnostics have increased pathogen detection, particularly in gastrointestinal and respiratory samples, and inevitably this has increased the detection of co-infections. In many cases the clinical implications of these co-infections have been hard to establish. Reanalysis of the Global Enteric Multicentre study, using molecular detection and quantification by qRT PCR, found that half of cases had more than one pathogen detected at a diarrhoea-associated load [55^▪▪^]. *Shigella* spp. and rotavirus, were most frequently detected as sole pathogens in diarrhoea-associated quantities, meaning that they were often true pathogens. Many other pathogens were not detected in diarrhoea-associated quantities or were associated with diarrhoea only in combination with other pathogens with stronger causal relationships. Therefore, simple detection of co-infection is not enough to understand its consequences, and even with quantification attribution is difficult.

Similar results come from studies of respiratory viruses in children: multiple viruses are often detected but few are consistently associated with disease. In a recent study of children with acute respiratory infection, 82% had a respiratory virus detected, 59% had a single virus, and 23% had co-infections [56^▪^]. Detection of multiple respiratory viruses was not associated with any difference in severity or outcomes compared to monoinfections in this population. However, in longer follow-up studies asthma features were more common in 6–8-year-old children with a previous admission with multiple respiratory viruses when compared with those with single respiratory viruses, even when accounting for age at previous admission [57]. We do not yet know whether co-infection causes later asthma symptoms or acts as a marker of those who are susceptible.

Despite limited evidence that co-infections between different respiratory viruses are important, there is a well established association between respiratory viral and bacterial co-infection which corresponds with more severe illness [58^▪▪^]. Beyond simple additive effects, mechanistic evidence comes from studying influenza and pneumococcal co-infections. Influenza often precedes pneumococcal pneumonia, at least partly because it causes a depletion alveolar macrophages which allows a smaller inoculum of bacteria to establish productive infection [59^▪▪^]. Interestingly, the extent of depletion of alveolar macrophages and consequent severity of the bacterial co-infection may be exacerbated by preexisting host factors such as obesity [60^▪^].

### Pathogen as host for co-infections

Co-infection usually implies two or more pathogens infecting the human (or animal) host, but nature is full of surprises and one clinically important type of co-infection turns out to involve viral infection of the principal pathogen. Leishmania parasites infected with endosymbiont Leishmania RNA virus 1 are more virulent in rodent models [61] and human patients [62^▪^,63^▪^] because the virus induces type 1 interferon production in host macrophages, impairing intracellular killing of Leishmania [64^▪▪^]. Targeting viral clearance improves cure in mice suggesting a potential therapeutic avenue for humans with this disease [65^▪▪^,66^▪^].

## CONCLUSION

Accumulating evidence indicates that co-infections frequently do matter, but it is often difficult to predict how and when they matter. The examples highlighted in this review illustrate the potential complexity of interactions between infections and their effects on the host. It is also clear that studying co-infections is challenging, particularly in the context of natural infection. Conflicting results and conclusions from the study of the same infections serve to illustrate that there must be many as yet unknown factors involved. The interactions between the blurred boundaries of infection and colonization will undoubtedly need to be considered in the future. Perhaps a complete understanding of the relevance of co-infections will only come when large-scale unbiased approaches like metagenomic sequencing are applied longitudinally and in conjunction with other omics approaches which characterize the host and microbiome, and are interpreted with machine-learning strategies rather than standard clinician classifications. For now, animal models and human challenge studies may offer an intermediate step for identifying specific pathogen–pathogen interactions.

## Acknowledgements

None.

### Financial support and sponsorship

A.J.C. receives funding from the UK Medical Research Council (MRC) and the UK Department for International Development (DFID) under the MRC/DFID Concordat agreement and is also part of the EDCTP2 programme supported by the European Union (MR/L006529/1).

### Conflicts of interest

There are no conflicts of interest.

## REFERENCES AND RECOMMENDED READING

Papers of particular interest, published within the annual period of review, have been highlighted as:

▪ of special interest
▪▪ of outstanding interest
